# Xanthohumol-Induced Rat Glioma C6 Cells Death by Triggering Mitochondrial Stress

**DOI:** 10.3390/ijms22094506

**Published:** 2021-04-26

**Authors:** Shaozhi Hou, Yang Song, Di Sun, Shujun Zhu, Zhenhua Wang

**Affiliations:** Center for Mitochondria and Healthy Aging, College of Life Science, Yantai University, Yantai 264005, China; ee916311@gmail.com (S.H.); songyang9611@gmail.com (Y.S.); sundi1128@gmail.com (D.S.); zhushujun2020@gmail.com (S.Z.)

**Keywords:** xanthohumol, mitochondrial stress, glioma cell

## Abstract

AIM: To investigate the underlying mechanisms of xanthohumol (XN) on the proliferation inhibition and death of C6 glioma cells. METHODS: To determine the effects of XN on C6 cells, cell proliferation and mortality after XN treatment were assessed by SRB assay and trypan blue assay respectively. Apoptotic rates were evaluated by flowcytometry after Annexin V-FITC/PI double staining. The influence of XN on the activity of caspase-3 was determined by Western blot (WB); and nuclear transposition of apoptosis-inducing factor (AIF) was tested by immunocytochemistry and WB. By MitoSOX^TM^ staining, the mitochondrial ROS were detected. Mitochondrial function was also tested by MTT assay (content of succinic dehydrogenase), flow cytometry (mitochondrial membrane potential (MMP)—JC-1 staining; mitochondrial abundance—mito-Tracker green), immunofluorescence (MMP—JC-1 staining; mitochondrial morphology—mito-Tracker green), WB (mitochondrial fusion-fission protein—OPA1, mfn2, and DRP1; mitophagy-related proteins—Pink1, Parkin, LC3B, and P62), and high-performance liquid chromatography (HPLC) (energy charge). Finally, mitochondrial protein homeostasis of C6 cells after XN treatment with and without LONP1 inhibitor bortezomib was investigated by trypan blue assay (proliferative activity and mortality) and WB (mitochondrial protease LONP1). All cell morphology images were taken by a Leica Microsystems microscope. RESULTS: XN could lead to proliferation inhibition and death of C6 cells in a time- and dose-dependent manner and induce apoptosis of C6 cells through the AIF pathway. After long incubation of XN, mitochondria of C6 cells were seriously impaired, and mitochondria had a diffuse morphology and mitochondrial ROS were increased. The content of succinic dehydrogenase per cell was significantly decreased after XN insults of 24, 48, and 72 h. The energy charge was weakened after XN insult of 24 h. Furthermore, the MMP and mitochondrial abundance were significantly decreased; the protein expression levels of OPA1, mfn2, and DRP1 were down-regulated; and the protein expression levels of Pink1, Parkin, LC3B-II/LC3B-I, and p62 were up-regulated in long XN incubation times (24, 48, and 72 h). XN incubation with bortezomib for 48 h resulted in lower proliferative activity and higher mortality of C6 cells and caused the cell to have visible vacuoles. Moreover, the protein expression levels of LONP1 was up-regulated gradually as XN treatment time increased. CONCLUSION: These data supported that XN could induce AIF pathway apoptosis of the rat glioma C6 cells by affecting the mitochondria.

## 1. Introduction

Glioma is one of the most common central nervous system (CNS) tumors. According to 2007 WHO CNS tumor classification, glioma is divided into five categories, including oligoastrocytic, oligodendroglial, astrocytic, ependymal, neuronal, and mixed neuronal–glial [1]. Recently, in 2016, WHO introduced a new edition of glioma classification, based on the degree of malignancy. Among all the types of glioma, glioblastoma is considered the most malignant, as it is classified as WHO grade IV [2,3]. The standard treatment for this tumor is surgery followed by radiotherapy and chemotherapy. To some extent, this treatment can extend patients’ survival time, but it can also cause many severe side effects that are undesirable. Mounting evidence shows that natural medicinal compounds could play diversified beneficial roles in cancer therapy, while with less adverse reaction [4,5,6].

**Figure 1:**
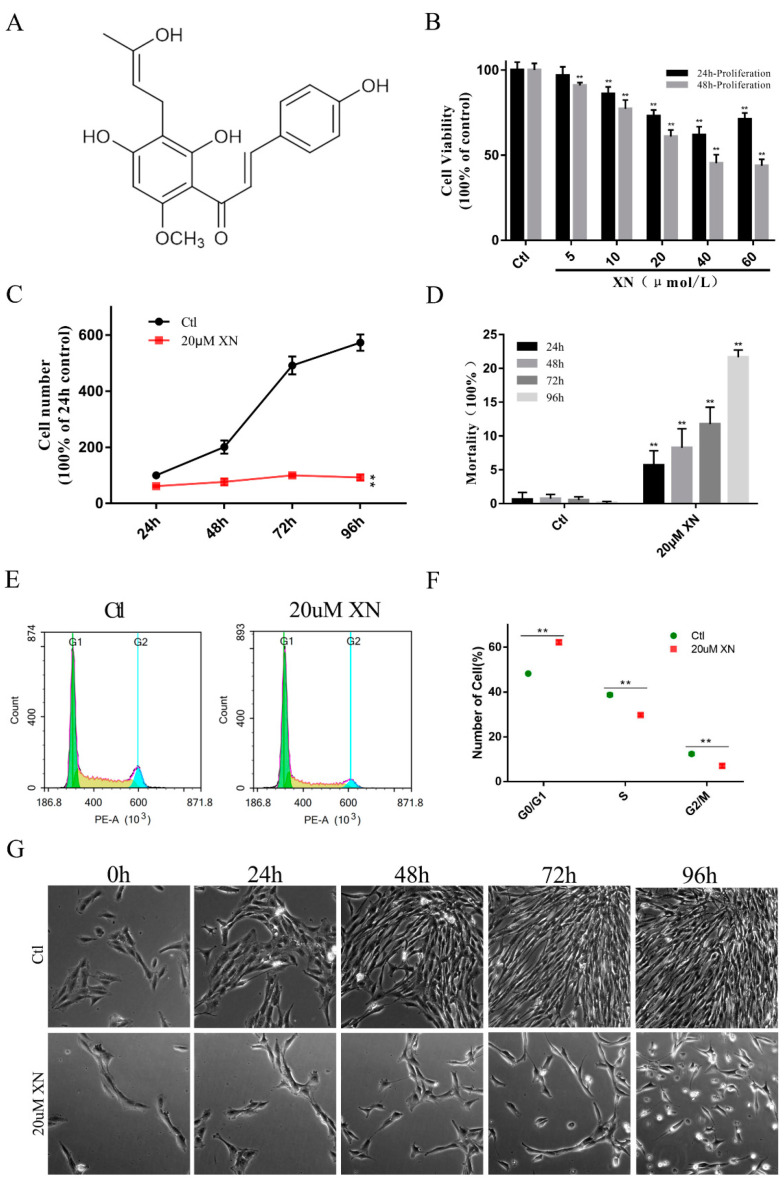
Effects of XN on proliferation activity and morphology of C6 cells. (**A**) The formula for XN. (**B**) The C6 cells were treated with XN for indicated concentration and time, and the cell viability was measured by SRB assay. (**C**,**D**,**G**) C6 cells were incubated for different time with 20 uM XN, the survived and dead cells were detected by trypan blue staining, and the morphology changes were captured by a Leica Microsystems microscope (200× *g* magnification). (**E**,**F**) The cell cycle of C6 cells treated with 20 uM XN for 48 h was detected by flow cytometry after PI mono-staining. All data presented are the mean ± SEM from three independent experiments. ** *p* < 0.01 vs. control groups.

Xanthohumol (XN) (Figure 1A), a natural compound consisted in hops, is an isoprene flavonoid with a wide range of biological activities, such as anti-inflammation, antioxidant, anti-cancer, antibacterial, and lipid-lowering benefits [7]. Several studies showed the anti-glioma effects of XN. Chen et al. found that the miR-204-3p-targeted IGFBP2 pathway was involved in XN-induced glioma cell apoptotic death [8]. And Festa et al. reported that XN induces apoptosis in human malignant glioblastoma cells by increasing reactive oxygen species (ROS) and activating MAPK pathways [9]. However, most of these studies investigated the mitochondria as the key node in the process of apoptosis but did not reveal the status of the mitochondria [8,9,10]. Therefore, in this study, we used an in vitro cultured rat glioma C6 cells to investigated the effects of XN on the cell survival and the mitochondrial response mechanism.

## 2. Results

Based on our research, it was confirmed that XN can inhibit C6 cell proliferation and eventually induce cell death by triggering mitochondrial stress. This is discussed in further detail in the following sections.

### 2.1. XN Affected C6 Cell Proliferation in Concentration and Time-Dependent Way 

Our primary data showed that XN significantly inhibited the C6 glioma cell proliferation in concentration and time-dependent way. Figure 1B clearly shows that with the increase of the concentration of XN from 5 uM to 60 uM, the cell viability decreased obviously. Along with the increase of the XN (20 uM) treatment time, the cell proliferation was completely suppressed (Figure 1C), while the cell mortality increased correspondingly (Figure 1D). Surprisingly, in this process, XN did not induce the obvious changing of cell morphology which allows us to check for more details about the death process of C6 cells. Therefore, this concentration was chosen for further investigation. Moreover, incubation for 48 h with 20 uM XN dramatically increased the cells in G0/G1 cycle phase (Figure 1E,F).

Overall, the data we collected strongly supported our expectations. XN treatment can certainly inhibit C6 cell proliferation and even cause its death when the treatment time is long enough.

### 2.2. XN Treatment-Induced AIF-Dependent Apoptosis in C6 Cells

**Figure 2:**
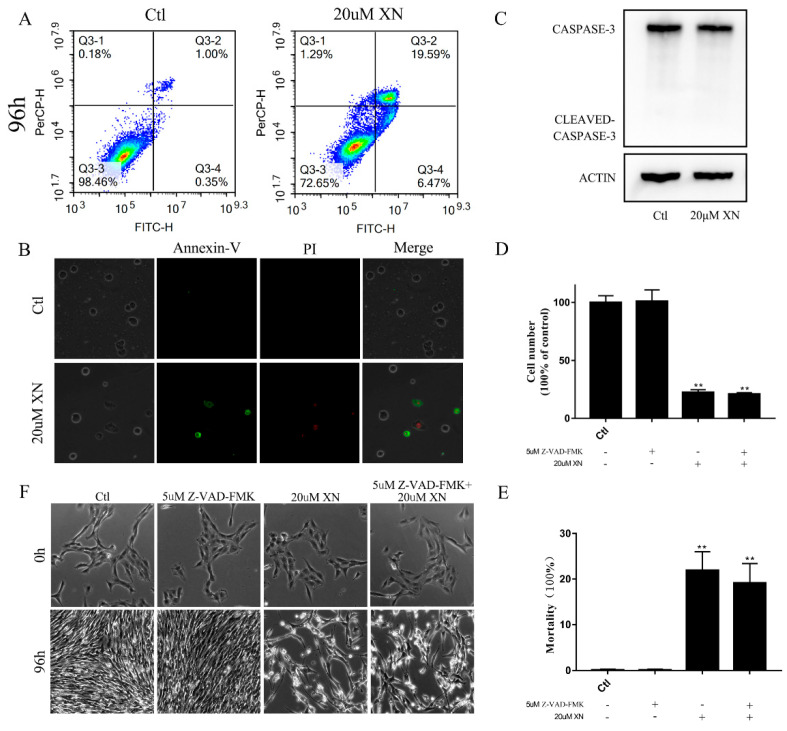
XN-induced apoptosis of C6 cells. (**A**,**B**) C6 cell apoptosis evaluation by flow cytometry and fluorescence microscopy through Annexin-V/PI double staining after treatment with XN for 96 h (400× *g* magnification). (**C**) Cleaved caspase-3 was detected by Western blot (WB) after XN incubation for 96 h. (**D**–**F**) The C6 cells were pretreated with 5 uM inhibitor of caspase-3 Z-VAD-FMK for 1.5 h before the co-treatment with XN for another 96 h. The morphology changes were captured by a Leica Microsystems microscope (200× *g* magnification), and cell viability and cell death were measured by trypan blue staining. All data presented are the mean ± SEM from three independent experiments. ** *p* < 0.01 vs. control.

**Figure 3:**
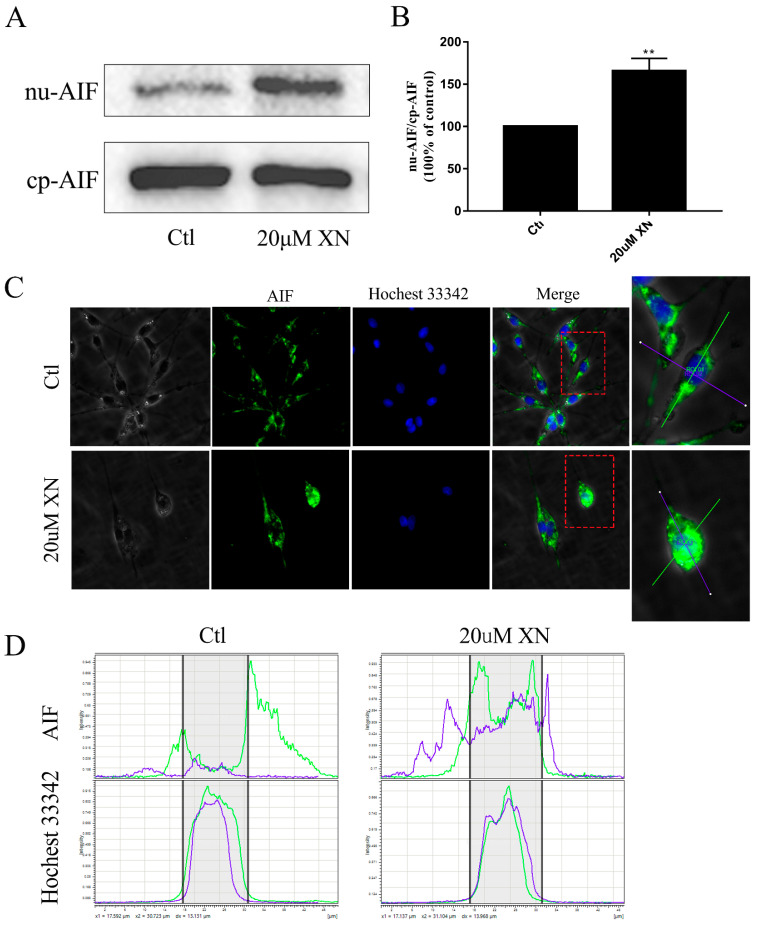
Effects of XN (20 uM) on intracellular localization of AIF. (**A**,**B**) The C6 cells were exposed to XN for 96 h. The levels of AIF in cytoplasm (cp-AIF) and nucleus (nu-AIF) were detected by WB. (**C**,**D**) Cells were treated with XN for 96 h, and then cells were subjected to immunocytochemistry using anti-AIF (green). Positioning was captured, and the fluorescence distribution was analyzed by a Leica Microsystems microscope (400× *g* magnification). All data presented are the mean ± SEM from three independent experiments. ** *p* < 0.01 vs. control groups.

In our study, XN treatment caused C6 cell apoptosis, and more specifically, *via* AIF-dependent pathway apoptosis [11,12,13]. Figure 1G shows the morphology changes of C6 cell at different times after 20 uM XN treatment. When C6 cells were treated with XN for 72 h, C6 cells began to shrink. After 96 h treatment with XN, more apoptotic cells were identified with the flowcytometry and fluorescence microscope observation compared to the control group (Figure 2A,B), while no Caspase-3 was cleaved as shown in Figure 2C. Moreover, treatment with the pan-caspase inhibitor Z-VAD-FMK did not show any improvement in cell viability (Figure 2D–F). Thus, we targeted AIF intracellular localization. AIF is a mitochondrial resident protein that regulates cell apoptosis via nuclear translocation [13]. The Western blot and fluorescence immunohistochemistry results showed that AIF content in nucleus increased significantly (Figure 3A,B) and a mass of AIF located in the nucleus after XN treatment (Figure 3C,D). To conclude, all the evidence we found can certainly prove that the XN-triggered C6 cell apoptosis pathway is AIF rather than cleaved caspase-3.

### 2.3. XN Increased the Amount of Mitochondrial ROS in the Process of Cell Death

**Figure 4:**
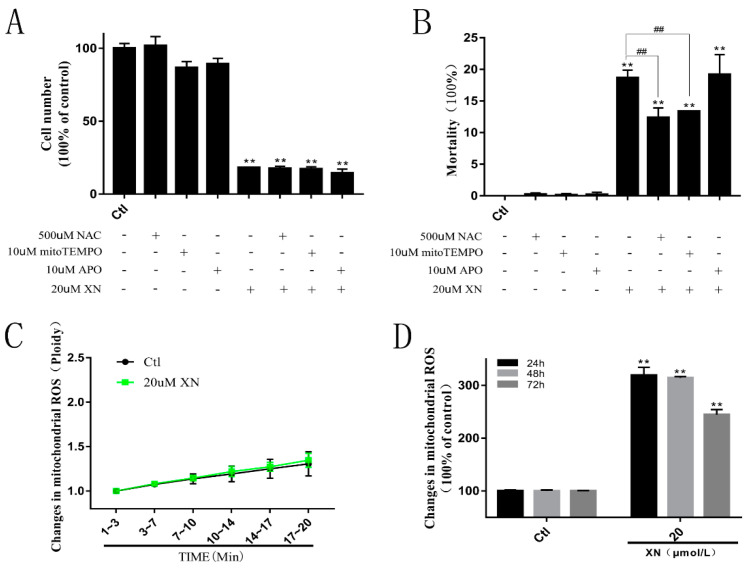
Effects of XN (20 uM) on ROS formation in C6 cells. (**A**,**B**) The C6 cells were pretreated with 500 uM NAC to scavenge the intracellular total ROS, or 10 uM Mito-Tempo to scavenge the mitochondrial ROS, or 10 uM APO to inhibit the NADPH oxidase for 1.5 h before the co-treatment with XN for another 96 h, and the cell viability and death were measured by trypan blue assay. (**C**) The change of mitochondrial ROS in C6 cells during the initial 20 min of XN incubation was detected by flow cytometry. (**D**) The change of mitochondrial ROS in C6 cells during the 24, 48, and 72 h of XN incubation was detected by flow cytometry. Mitochondrial ROS were labeled by mitoSOX^TM^. ** *p* < 0.01 vs. control groups. All data presented are the mean ± SEM from three independent experiments. ## *p* < 0.01 vs. XN-treated group.

Reactive oxygen species as cell signaling molecules play very important roles in cell metabolic processes. The generation of ROS can protect the cells and also help send signals in normal cell biological processes. However, apart from the benefits to cells, imbalanced production and accumulation of ROS can damage the cells *via* oxidative stress, and eventually cause pathophysiological changes, such as apoptosis [14,15,16,17]. Referring to Section 2.2, the incubation of XN can cause C6 cell apoptosis, and it can be predicted that cell death caused by XN treatment may be related to the increase of ROS. An investigation which involved three antioxidants (NAC, Mito-Tempo, or APO) was undertaken to explore the ROS formation in XN induced cell death. As expected, the results showed that when NAC or Mito-Tempo were mixed with XN in the solution, fewer C6 cells died despite continuous exposure to proliferation inhibition, but when APO was used, there was no change in cell death rates (Figure 4A,B). In addition, Figure 4C,D show that XN did increase the amount of ROS in C6 cells; and the number went up to the top when C6 cells were incubated by XN for 24 h and 48 h. Thus, it can be concluded that XN can kill C6 cell by increasing mitochondrial ROS.

### 2.4. XN Affected Mitochondrial Metabolism in C6 Cells

**Figure 5:**
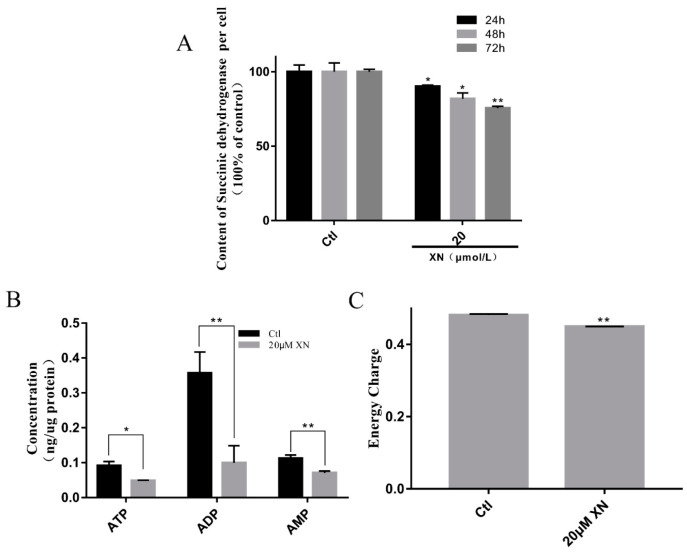
Effect of XN on energy metabolism of C6 cells. (**A**) MTT assay was used to assay the effects of XN treatment on C6 cells within different times. (**B**,**C**) XN (20uM) reduced the ATP content and energy charge. Cells were treated with XN (20uM) for 24 h, then the content of ATP, ADP, and AMP were determined by HPLC. All data presented are the mean ± SEM from three independent experiments. * *p* < 0.05, ** *p* < 0.01 vs. control groups.

Mitochondria is a relatively independent organelle in cells, with its own genome. It is the place where cell energy comes from and plays a significant role in a cell’s life [18,19,20]. In order to further investigate if XN induced cell death by interfering with mitochondrial metabolism, we analyzed the mitochondrial metabolic activities by MTT assay in C6 cells. As well-known, MTT can interact with succinic dehydrogenase in mitochondria to form formazan [21,22]. Under the premise of the constant cell number, the formazan production is related to the mitochondrial function of cells. With the result shown in Figure 5A, XN treatment indeed impaired the mitochondrial function in time-dependent way. Further investigation of the intracellular contents of ATP, ADP and AMP with HPLC [23] proved that XN treatment just slightly decreased the cell energy charge (Figure 5C), while it significantly brought down the total content of ATP, ADP and AMP to 31.83% of control (Figure 5B). To summarize, our research proved that XN can affect the mitochondrial metabolism and reduce cell energy charge.

### 2.5. XN Reduced Mitochondrial Membrane Potential (MMP) in C6 Cell

**Figure 6:**
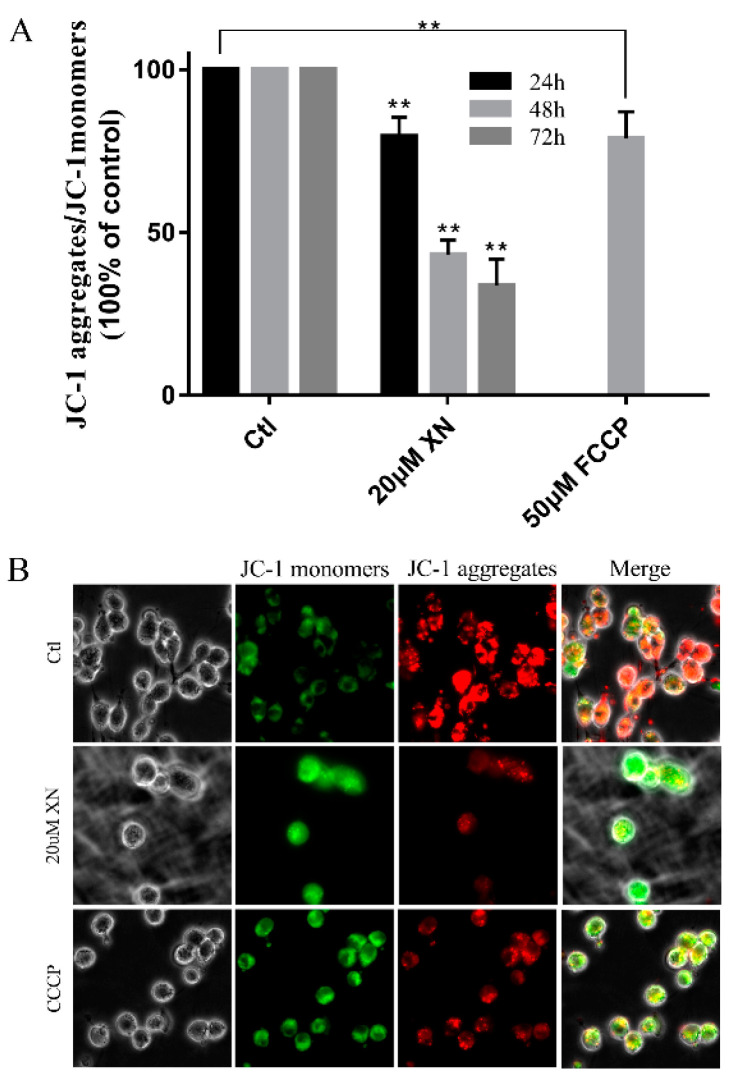
XN (20uM) affected the MMP of C6 cells. (**A**) The C6 cells were treated with XN for the indicated different times or 50μM FCCP for 2 h. The cells were incubated with JC-1 for 30 min before flow cytometry detection. (**B**) Cells were treated with 20 uM XN for 48 h or 100 μM CCCP for 30 min, then visualized by a Leica Microsystems microscope after being incubated with 10 μM JC-1 for 30 min (400× *g* magnification). All data presented are the mean ± SEM from three independent experiments. ** *p* < 0.01 vs. control groups.

As the key marker of mitochondrial function, the mitochondrial membrane potential is an essential driver of ATP synthesis. MMP is so important that its drops can lead to reduced mitochondrial productivity and even cell death [24,25,26,27]. In our study, flow cytometry and immunofluorescence analysis showed a decrease in JC-1 aggregates after C6 cells were incubated by XN (Figure 6). Thus, it can be seen that XN induced MMP decrease.

### 2.6. XN Caused Fusion and Fission Imbalance by Reducing the Mitochondrial Fusion Function in C6 Cells

**Figure 7:**
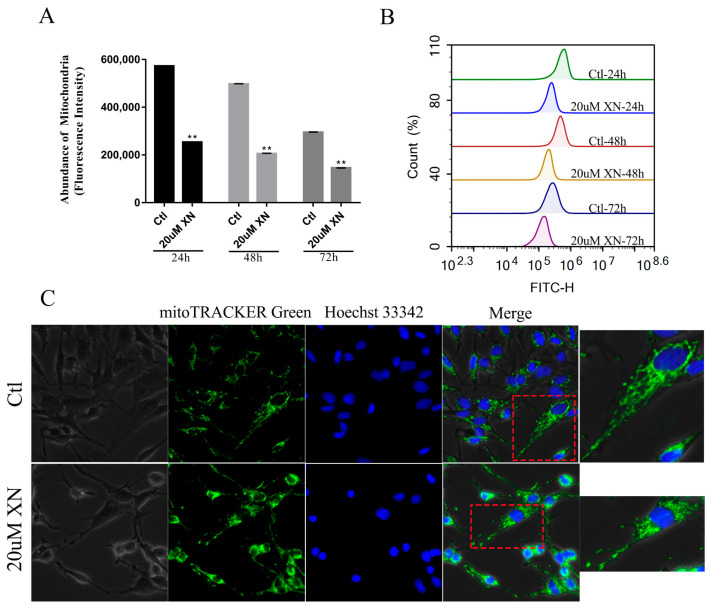
Effect of XN (20 uM) on mitochondrial abundance and mitochondrial morphology in C6 cells. (**A**,**B**) After treatment with XN for 24, 48, and 72 h, mitochondrial abundance was detected by flow cytometry after being incubated with 150 nM Mito-Tracker green for 30 min. (**C**) Cells were treated with XN for 48 h, and then visualized by a Leica Microsystems microscope after incubated with 150 nM Mito-Tracker green and 10 uM Hoechst 33342 for 30 min (400× *g* magnification). All data presented are the mean ± SEM from three independent experiments. ** *p* < 0.01 vs. control groups.

**Figure 8:**
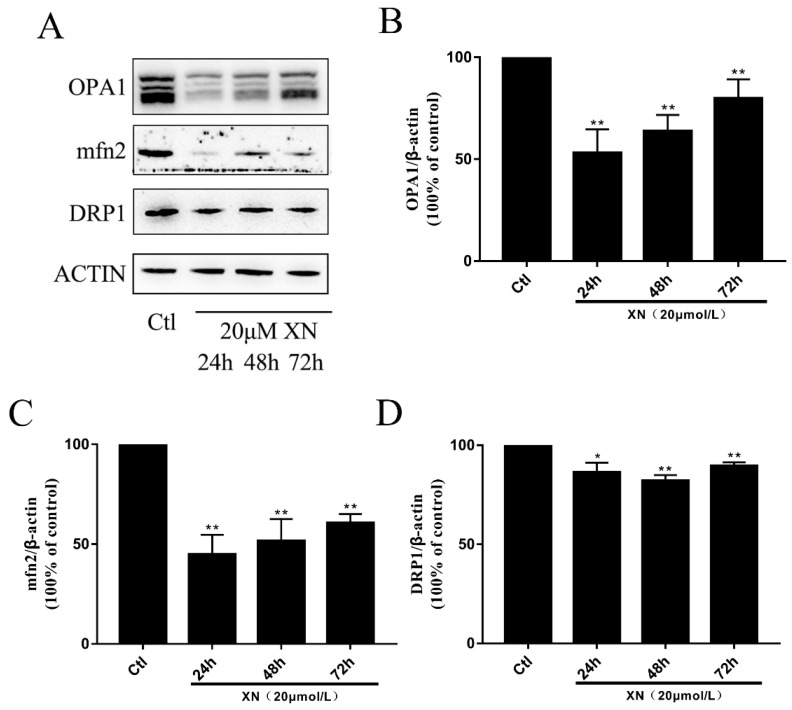
Effect of XN (20 uM) on mitochondrial fusion and fission. The C6 cells were exposed to XN for 24 h, 48 h, and 72 h. (**A**)The OPA1, mfn2, and DRP1 expression levels were analyzed by Western blot, and ACTIN was used as a loading control. (**B**–**D**) Quantitative analysis of the expression levels of OPA1, mfn2 and DRP1. All data presented are the mean ± SEM from three independent experiments. * *p* < 0.05, ** *p* < 0.01 vs. control groups.

In cells there is a dynamic balance to maintain the mitochondrial function for a cell’s normal life [28,29]. Mitochondrial fusion and fission are set in a suitable speed to keep the balance. This process is controlled by three key proteins, which are two fusion proteins (OPA1 and mfn2) and one fission protein (DRP1) [30,31]. Based on previous experiments, we concluded that XN treatment can cause mitochondrial dysfunction. However, it is unclear if XN triggers apoptosis through impairing the mitochondrial balance. Our further investigation indicated that the XN treatment decreased the intracellular abundance of mitochondria (Figure 7A,B) and kept the mitochondria in a fragmented state (Figure 7C). In the process of damaging the balance, the fission protein DRP1 just slightly dropped down (Figure 8A,D), whereas the OPA1 and mfn2 decreased dramatically. Although the level of those protein went up slightly when XN treatment time was increased, overall the cells treated with XN were disturbed significantly, and mitochondrial fusion-fission balance was impaired seriously. Figure 8 have expressed the change clearly. To sum up, XN can certainly break down the balance by reducing the three proteins, especially the two fusion proteins, OPA1 and mfn2.

### 2.7. XN-Induced Mitophagy in C6 Cell

**Figure 9:**
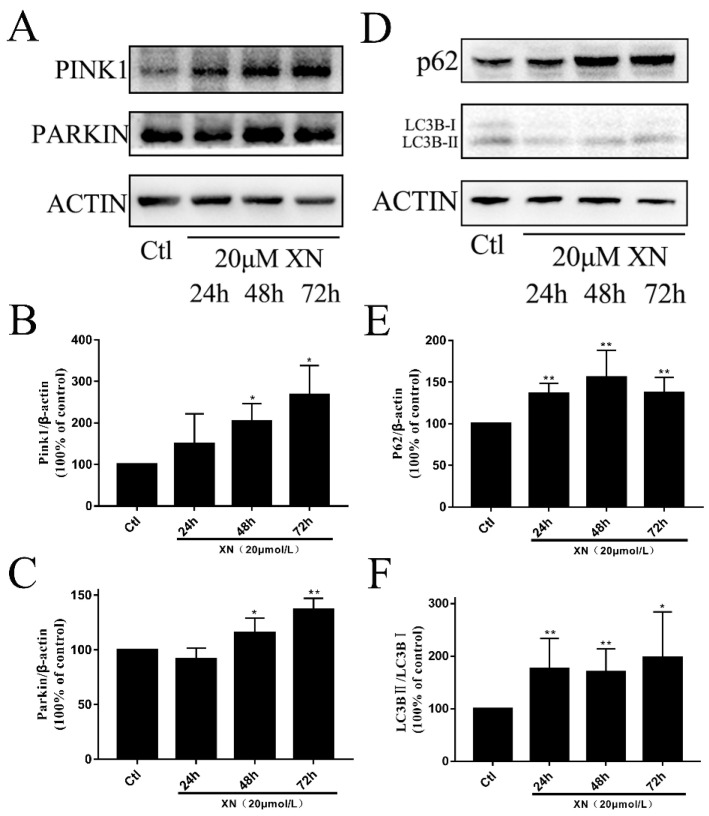
XN (20 uM)-induced mitophagy in C6 cells. (**A**–**C**) Cells were treated with XN for 24, 48, and 72 h, and then protein expression of PINK1, PARKIN was determined by Western blot analysis. (**D**–**F**) Cells were treated with XN for 24, 48, and 72 h, and then protein expression of LC3B, and p62 was determined by Western blot analysis. All data presented are the mean ± SEM from three independent experiments. * *p* < 0.05, ** *p* < 0.01 vs. control groups.

Mitophagy is a process that selectively degrades mitochondria that are damaged or in stress. It is one of the ways for cells to remove abnormal mitochondria and maintain cells’ healthy environments [32,33]. In cells, those mitochondria with low membrane potential or that are diffused tend to be removed through mitophagy in different pathways. For example, mammalian cells normally clear damaged mitochondria via the pink1-parkin pathway [34,35,36]. As is explained in 2.5, XN can reduce MMP in C6, and furthermore, mitochondrial morphology is presented in an unfussed state. Those mitochondria in unfussed states in C6 were the ones being cleared, and Figure 9A–C shows the obvious increase of PINK1 and PARKIN, and Figure 9D and 9F show the obvious raise of LC3B-II/LC3B-I. This is enough to prove that mitophagy occurred due to XN treatment.

In addition to triggering mitophagy, whether XN also has impacts on autophagy flow was also checked by analyzing the quantity of p62 protein, which is considered as a marker of normal fusion between autophagosomes and lysosomes [5,37,38]. Figure 9D,E show obvious increases of p62 in the XN group, which indicates that XN inhibited the fusion between autophagosomes and lysosomes. In a word, XN can induce mitophagy as well as affect autophagy flow to damage the cells ultimately.

### 2.8. LONP1 Alleviated the Injury of XN

As discussed before, XN can greatly affect mitochondrial function, including mitochondrial fusion dysfunction and lower MMP. To explore more details about how XN can cause this series of reactions, we further investigated the level of LONP1, a mitochondrial matrix protein that belongs to the Lon family of ATP-dependent proteases, which mediates the selective degradation of misfolded, unassembled or oxidatively damaged polypeptides in the mitochondrial matrix [39,40,41]. The level of LONP1 expression is regulated by a variety of cellular stresses, including unfolded protein stress [41,42,43]. In a word, LONP1 is supposed to be in a balanced level to maintain cell’s healthy and normal functions.

**Figure 10:**
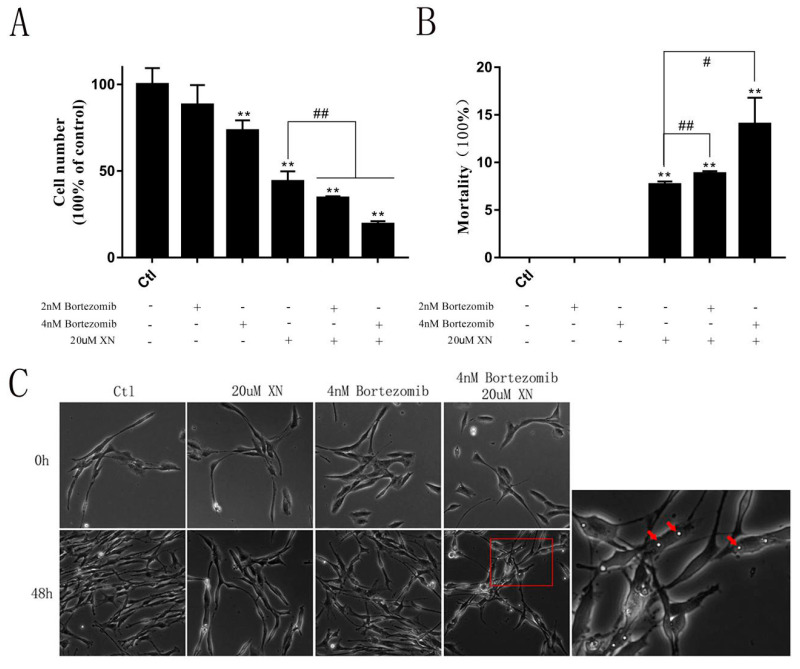
Effect of bortezomib on XN (20 uM)-induced inhibition of C6 cell proliferation. The C6 cells were pretreated with 2 nM or 4 nM bortezomib, and inhibitor of the LONP1 for 1.5 h before the co-treatment with XN for another 48 h. (**A**,**B**) The cell viability and the cell deaths were measured by SRB assay. (**C**) The morphology changes were captured by a Leica Microsystems microscope (200× *g* magnification), and intracellular vacuoles were marked by red arrows. All data presented are the mean ± SEM from three independent experiments. ** *p* < 0.01 vs. control groups. # *p* < 0.05, ## *p* < 0.01 vs. XN-treated group.

**Figure 11:**
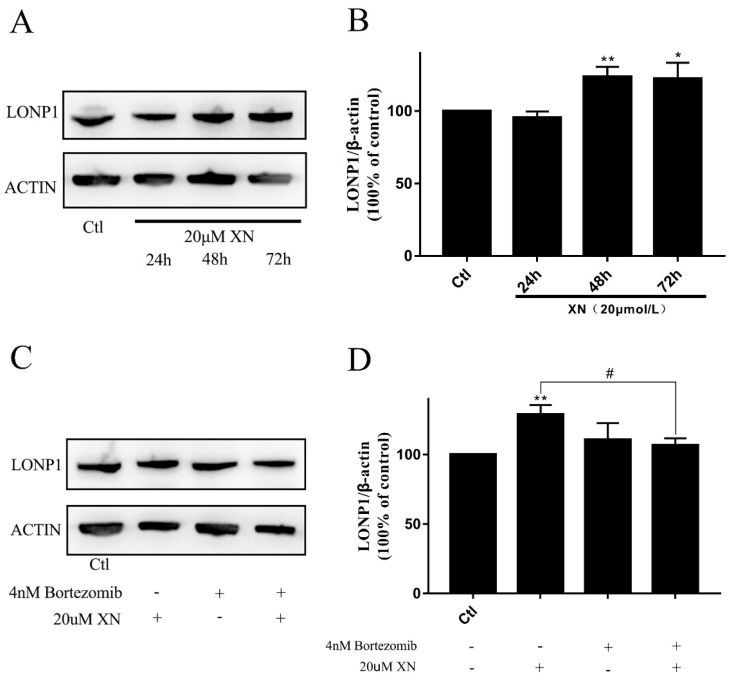
Effect of XN (20 uM) on the level of LONP1 protein with or without bortezomib. (**A**,**B**) Cells were treated with XN for 24, 48, and 72 h, and then protein expression of LONP1 was determined by Western blot analysis. (**C**,**D**) The C6 cells were pretreated with 4 nM bortezomib for 1.5 h before the co-treatment with XN for another 48 h, and then protein expression of LONP1 was determined by Western blot analysis. All data presented are the mean ± SEM from three independent experiments. * *p* < 0.05, ** *p* < 0.01 vs. control groups. # *p* < 0.05.vs the XN treated group.

In this study, although just a slightly increase of LONP1 protein was induced by XN exposure for at least 48 h (Figure 11A,B), the pretreatment with LONP1 inhibitor bortezomib obviously exacerbated the C6 cell proliferation inhibition and mortality as well as cytoplasmic vacuoles formation induced by XN exposed (Figure 10). Consistent with this, bortezomib pretreatment entirely reversed the LONP1 increase induced by XN (Figure 11C,D). That means LONP1 played a certain role in alleviating XN injury, though the changes in LONP1 expression were not so prominent. 

## 3. Discussion

XN, a natural compound, has been reported due to its anti-cancer benefits [7]. Recently, there have been many studies about how XN can treat glioma [8,9,10]. However, there are very few articles about the effects of XN on the mitochondria. Thus, this work focuses on how XN affects glioma C6 cells based on mitochondria. We firstly confirmed that XN can inhibit C6 proliferation and induced cell death in the concentration and time-dependent way. Then we proved that XN treatment blocked the cell cycle at G0/G1 phase and induced the AIF-mediated apoptosis, which was accompanied by the mitochondrial structure and function impairment and mitophagy blockage.

Mitochondria play important roles in maintaining the cellular endoenvironmental stability and modulating cell survival not only by producing ATP but also via releasing some modulators such as AIF and bcl-2 to control the cell fate [18,19,20,43,44]. In our study, the XN-induced mitochondrial impairments were identified by excess ROS formation, decrease of succinic dehydrogenase content and MMP, imbalance of fusion and fission, and the ATP synthesis. The XN-induced C6 cell death was partially reversed by the intracellular total ROS scavenger NAC and the mitochondrial ROS scavenger mito-TEMPO, which indicates the oxidative stress is involved in the cell death mediated by mitochondrial dysfunction. 

In order to maintain the mitochondrial healthy, the damaged mitochondria are cleared by mitophagy after their fission [34,35,36]. So, the dynamic equilibrium of fission and fusion of mitochondria is essential for cell survival even it needs to consume lots of energy. In addition, an ATP-dependent serine protease, LONP1 also contributes to the mitochondrial homeostasis by degradation of misfolded, unassembled or oxidatively damaged proteins in the mitochondrial matrix, which also needs to consume energy. So, the mitochondria injury not only impairs the cell ATP synthesis, but also consumes a large amount of ATP to restore intracellular stability. This vicious cycle exacerbates cellular energy depletion. Here our results showed XN-treatment induced a dramatically decrease of the intracellular ATP content at the early stage of XN exposure, which further supported the mitochondrial dysfunction played key role in XN-induced cell death. However, the exact underlying mechanism and the target of XN involved in mitochondrial stress needs to be further elucidated.

As discussed before, the mitochondrial environment was disturbed badly after XN treatment, and XN also blocked the main clear pathway of mitochondria-the mitophagy effectively, so C6 cells could not do more to reverse the damage from XN, and finally went to their last stage-apoptosis, which is the most common type of cell death [11,12]. Furthermore, our study explained more specifically that C6 cell apoptosis happened via the AIF pathway instead of through the caspase-3 pathway. Although much more work will need to be done before XN truly becomes the clinical treatment for glioma, our study on rat glioma C6 cells has provided a very positive start and will become a very supportive reference for future XN research on human glioma cells.

In summary, as a natural compound, XN may be a good source of glioma treatment through triggering mitochondrial stress and the AIF-mediated apoptosis in glioma.

## 4. Materials and Methods

### 4.1. Chemicals and Reagents

XN was purchased from Nanjing Spring & Autumn Biological Engineering Co. Ltd. The purity of XN was over 99% assayed by HPLC. XN was stored in dimethyl sulfoxide (DMSO) at a concentration of 100 mM at −20 °C and diluted to the required concentration before use. The 0.4% trypan blue reagent was bought from Solarbio Life Science (Beijing, China). N-Acetyl-L-cysteine (NAC) was bought from Beyotime (Shanghai, China). Mito-Tempo was bought from Sigma-Aldrich (St. Louis, MO, USA). Bortezomib was purchased from Sigma-Aldrich. Apocynin (APO) was purchased from Selleck (Houston, TX, USA). Z-VAD-FMK was purchased from Selleck.

### 4.2. Cell Culture

Rat glioma C6 cell was provided by the Shanghai Cell Bank (Shanghai, China). The cells were cultured by F12 (Gibco, Waltham, MA, USA) supplemented with 5% (*v*/*v*) fetal calf serum (FBS) (TIANHANG) and 5% (*v*/*v*) horse serum (HS) (Solarbio), 100 U/mL penicillin, and 100 mg/mL streptomycin (Gibco). The C6 cells were cultured in a 5% CO_2_, 37 °C humidified incubator and were passaged once every two days. The experiments were classed as control group (containing DMSO, 0.1% DMSO) and experimental group (containing compounds). According to different conditions, cells were seeded (3000 cells/cm^2^) into different culture plates in the exponential growth phase.

### 4.3. Cell Viability Assay and Morphological Observation

The proliferative activities of C6 cells with XN incubation were evaluated by the SRB assay. C6 cells were seeded in 96-well microplates and cultured for 48 h. Then, cells were treated with different doses of XN for 24 h and 48 h. Next, 50% trichloroacetic acid was added to the 96-well microplates (final concentration: 10%). After incubation for 1 h at 4 °C, the cells were washed with ultrapure water and then dried. Next, cells were stained with 0.4% SRB for 20 min at room temperature. Furthermore, cells were then washed with 1% glacial acetic acid. After washing and airing, bound SRB was dissolved in 100 μL of 10 μM unbuffered Tris base. The absorbance was measured at 540 nm using a microplate reader (Molecular Devices, Sunnyvale, CA, USA). For cell morphology, cell photos were captured by a Leica Microsystems microscope (Solms, Germany).

### 4.4. Trypan Blue Assay

After XN treatment, all the cells were recovered and made into cell suspensions, and cell suspensions were mixed with 0.4% trypan blue solution at room temperature (cell suspension volume:solution volume = 9:1). Each count was done within three minutes using a hemocytometer.

### 4.5. Cell Cycle Assay

C6 cells were seeded in 6-well plates for 48 h and then treated with different treatments for indicated times. The cell suspension was then prepared and stained with a Cell Cycle and Apoptosis Analysis Kit (Beyotime Biotechnology, Shanghai, China) according to the instruction book. Cell Cycle flow cytometry was carried out on a Novocyte Instrument using Novo Express software (ACEA, China).

### 4.6. Apoptosis Assay

C6 cells were seeded in 6-well plates for 48 h and then treated with different treatments for indicated times. The cell suspension was then prepared and stained with an Annexin V-FITC Cell Apoptosis Assay Kit (Beyotime Biotechnology, Shanghai, China) according to the instruction book. Apoptosis flow cytometry was carried out on a Novocyte Instrument using Novo Express software (ACEA, China), and the stained cells were examined by a Leica Microsystems microscope.

### 4.7. Measurement of Mitochondrial ROS

MitoSOX^TM^ (ThermoFisher, Massachusetts, America) was used to measure the mitochondrial ROS formation. In brief, logarithmic growth phase cells were taken to make cell suspensions, and then the 0.3 mL cell suspension containing 1.3 × 10^5^ cells were selected and fused with 0.1 mL 20 μM MitoSOX^TM^. After 0.5 h incubation, compounds of different concentrations (final volume: 1 mL) were added, and the fluorescence was continuously monitored for 20 min by flow cytometry.

Cells were seeded in 6-well plates. Then, after 48 h, cells were treated with 20 μM XN for another 24 h, 48 h, or 72 h, followed by incubation with 5 μM MitoSOX^TM^ at 37 °C for 30 min in the incubator. The MitoSOX^TM^ signal was visualized by flow cytometry.

### 4.8. Detection of the Mitochondrial Membrane Potential

Cells were seeded in 6-well plates. Then, after 48 h, cells were treated with 20 μM XN for another 24 h, 48 h, or 72 h or 50 μM FCCP (Sigma-Aldrich) for another 2 h, followed by incubation with 10 μM JC-1 (Sigma-Aldrich) at 37 °C for 30 min in the incubator. The JC-1 signal was visualized by flow cytometry.

Cells were seeded on coverslips in 24-well plates. Then, after 48 h, cells were treated with 20 μM XN for another 48 h or 100 μM CCCP (Sigma-Aldrich) for another 30 min, followed by incubation with 10 μM JC-1 at 37 °C for 30 min in the incubator. The JC-1 signal was tested by a Leica Microsystems microscope.

### 4.9. Western Blot Analysis

After XN incubation, all the C6 cells were collected in 1.5 mL Eppendorf tube (EP tube), and RIPA buffer was used with protease inhibitor PMSF to lyse the cells on ice for 20 min. Then, lysates were centrifuged at 12,000× *g* for 20 min at 4 °C. The supernatant was collected to another EP tube. Furthermore, the nucleus proteins were obtained by a nuclear and cytoplasmic protein extraction kit (Beyotime Biotechnology). Then, the concentration of the protein samples was tested by an enhanced BCA protein assay kit (Beyotime Biotechnology); and about 30 μg denatured protein was separated using 6%, 10%, 12%, or 15% SDS-PAGE gel, and then the protein was transferred onto PVDF membranes. Membranes were blocked in 5% skimmed milk for 1 h at room temperature. Then the PVDF membranes were washed three times with TBST (10 min once time), incubated with primary antibodies (anti-caspase-3, anti-LC3B, anti-p62, anti-Pink1, anti-Parkin, anti-Drp1, anti-mfn2, anti-OPA and anti-LONP1) at 4 °C overnight, and the next day the membranes were washed three times (10 min each time) with TBST. Subsequently, the membranes were incubated with horseradish peroxidase (HPR)-conjugated secondary antibodies for 1 h at room temperature. Membranes were washed and incubated with ECL reagents, and finally the membranes were visualized by a 5200 multi luminescent image analyzer (Tanon Science & Technology Co., Ltd. Shanghai, China). ImageJ software was used to quantify the intensity of immunoreactive bands and normalized to loading controls. Anti-caspase-3, anti-p62, anti-Pink1, anti-Parkin, anti-Drp1, anti-mfn2, anti-OPA1, and anti-LONP1 were purchased from Cell Signaling Technology (Massachusetts, America). Anti-LC3B was purchased from Abcam (Cambridge Science Park, UK).

### 4.10. Immunocytochemistry

After treatment with XN, cells seeded on coverslips were washed three times with PBS, then fixed with 4% paraformaldehyde for 15 min and washed three times with PBS; and permeabilized with 0.5% PBS-Triton X-100 for 15 min and washed three times with PBS; and then incubated with 5% BSA (free fatty acids) for 30 min and washed three times with PBS. Then, cells were incubated with anti-AIF overnight at 4 °C. The next day, cells were washed three times with PBST, followed by FITC-labeled secondary antibody (1:500, Beyotime, China) for 1 h at room temperature. Hoechst 33342 was used to stain cell nuclei at a concentration of 10 μM for 20 min. Images were taken and observed using a Leica Microsystems microscope.

### 4.11. Detection of ATP, ADP, and AMP

C6 cells were seeded in 100 mm culture dishes. After 48 h, cells were incubated with XN for 24 h. Then, all cells were collected and divided into the same two parts to EP tubes—one to detect the protein concentration, another to detect the levels of ATP, ADP, and AMP. The concentration of the protein samples was determined by an enhanced BCA protein assay kit, and the levels of ATP, ADP, and AMP were detected by HPLC. First, cells were resuspended with 450 μL 5% cold perchlorate and were broken by repeated freeze–thaw cycles in liquid nitrogen (5 times). Then, the mixture was centrifuged at 14,000× *g* for 10 min at 4 °C. A total of 400 μL supernatant was collected, and the steps above were repeated, and a total of 350 μL supernatant was collected. Then the pH was adjusted to neutral using 1 mM KOH solution, and the final volume was recorded in detail. The analytical column was a SUPELCO ODS C18 column (250 mm × 4.6 mm, 5 μm). The mobile phase was 0.1M phosphate buffer, and the flow rate was set to 0.6 mL/min. The level of ATP, ADP, and AMP was determined by an adjustable UV-detector at 254 nm. The energy charge: ([ATP] + 1/2[ADP])/([ATP] + [ADP] + [AMP])

### 4.12. Detection of Content of Succinic Dehydrogenase

Cells were seeded in 24-well plates. After 48 h, cells were incubated with XN for 24, 48, and 72 h, and the content of succinic dehydrogenase was determined by MTT assays. Then 10% MTT (3-(4,5-dimethylthiazol-2-yl)-2,5-diphenyltetrazolium bromide) (Beyotime Biotechnology, Shanghai, China) (5 mg/mL) was added (60 μL per well) and then incubated for 2–4 h in the incubator. After formazan crystals were sufficiently dissolved by DMSO, the optical density (OD) at 490 nm was measured using the Multimode Detection platform (Molecular Devices, San Jose, CA, USA). Furthermore, the number of living cells passing through the Trypan blue assay was counted. Succinate dehydrogenase content was calculated with the same number of living cells.

### 4.13. Immunofluorescence staining

C6 cells were grown on coverslips in 24-well plates. After treatment with XN for 48 h, cells were incubated with 150 nM Mito-Tracker green and 10 μM Hoechst 33342 for 30 min at 37 °C. The fluorescence signal was visualized by a Leica Microsystems microscope.

### 4.14. Detection of Mitochondrial Abundance

C6 cells were seeded in 6-well plates. After treatment with XN for 24, 48, and 72 h, cells were incubated with 150 nM Mito-Tracker green for 30 min at 37 °C. The fluorescence signal was visualized by flow cytometry.

### 4.15. Statistical Analysis

Data were expressed as mean ± SEM of at least three independent experiments. Differences between groups were analyzed using non-paired Student’s t-test by SPSS 18.0 software (SPSS Inc., IBM Company, Chicago, IL, USA). Differences between groups were considered statistically significant at *P* < 0.05. Furthermore, all analyses were performed using GraphPad Prism 5 (GraphPad Software Inc., San Diego, CA, USA).

## 5. Conclusions

In conclusion, glioma, as one of the most malignant CNS tumors with poor prognosis, currently has only the standard treatment of surgery followed by chemotherapy and radiotherapy. This traditional treatment has many severe side effects, so we eagerly need to find a new treatment for those patients who are suffering from glioma. This study proves that XN can induce rat glioma C6 cells death by triggering the mitochondrial stress. The results are very positive indications that XN may possibly benefit cancer treatment in the future, for its induction of cell apoptosis via activating AIF pathway, which might be complementary to the classical apoptotic pathway dependent on caspase-3.

## Data Availability

Data sharing not applicable.
